# Transient dynamics in trial-offer markets with social influence: Trade-offs between appeal and quality

**DOI:** 10.1371/journal.pone.0180040

**Published:** 2017-07-26

**Authors:** Edgar Altszyler, Franco Berbeglia, Gerardo Berbeglia, Pascal Van Hentenryck

**Affiliations:** 1 Departamento de Computación, Universidad de Buenos Aires and CONICET, Buenos Aires, Argentina; 2 Tepper School of Business, Carnegie Mellon University, Pittsburgh, Pennsylvania, United States of America; 3 Melbourne Business School, University of Melbourne, Carlton, Victoria, Australia; 4 Industrial and Operations Engineering, University of Michigan, Ann Arbor, Michigan, United States of America; Universitat Jaume I, SPAIN

## Abstract

We study a trial-offer market where consumers may purchase one of two competing products. Consumer preferences are affected by the products quality, their appeal, and their popularity. While the asymptotic convergence or stationary states of these, and related dynamical systems, has been vastly studied, the literature regarding the transitory dynamics remains surprisingly sparse. To fill this gap, we derive a system of Ordinary Differential Equations, which is solved exactly to gain insight into the roles played by product qualities and appeals in the market behavior. We observe a logarithmic tradeoff between quality and appeal for medium and long-term marketing strategies: The expected market shares remain constant if a decrease in quality is followed by an exponential increase in the product appeal. However, for short time horizons, the trade-off is linear. Finally, we study the variability of the dynamics through Monte Carlo simulations and discover that low appeals may result in high levels of variability. The model results suggest effective marketing strategies for short and long time horizons and emphasize the significance of advertising early in the market life to increase sales and predictability.

## 1 Introduction

Social influence is a ubiquitous phenomenon in numerous markets: Product recommendations and information about past purchases have been shown to influence consumers choices significantly whether it is for music, movie, book, technological, and other type of products. Social influence often induces a rich-get-richer phenomenon (or Matthew effect) where popular products tend to become even more popular [1, 2]. Moreover, in many online platforms, this effect is amplified by product visibilities, since popular products appear in more prominent positions, further increasing purchases [3, 4]. Most social influence studies analyze the probability distribution of the market shares in the limit, using the well-known Pólya’s urn model [5, 6] and its variations. These model of product competition are a particular case of *cumulative advantage* processes [2, 7, 8].

In this paper, we consider a trial-offer market where consumers may purchase one of two competing products and consumer preferences are affected by the quality, appeal, and popularity of the products. While the asymptotic convergence of these, and related, dynamical systems has been widely studied (e.g., [9, 10]), the literature characterizing the transitory dynamics remains sparse. However, in many situations, the short-term dynamics is much more important than the asymptotic behavior, as the number of customers is finite. As an illustration, consider two firms *A* and *B* that compete for sales of their latest mobile devices. Each firm knows that its current mobile device will be out of the market in about two years and is not interested in a longer time horizon. Firm *A* may know that its device is of better quality and that, given enough time, it will capture the entire market share. But, in the short term (which may be two year long), firm *B* may try to compete with an inferior product through a large advertisement campaign which will induce early consumers to buy its product. These early adopters in turn will influence subsequent consumers to purchase *B*’s device through the social influence signal (e.g., the popularity of the products from *A* and *B*). As a result, it is particularly important to study the short-term dynamics for such trial-offer models.

This paper focuses on such trial-offer markets over a finite horizon and is particularly interested in answering the following questions to design a particular marketing campaign:

- Can a low quality be compensated by a greater appeal of the product?
- Given the quality and appeal of a competing product, which combination of quality and appeal are necessary for a product to capture the desired market share?
- How is important the timing of a marketing strategy?
- Will the marketing strategy increase or decrease the predictability of the market?

The main contribution of this paper is a principled study of these questions, to move from an understanding of the market behavior to the design of successful marketing and product development strategies. First, we derive a system of Ordinary Differential Equations (ODEs) which approximates the discrete market dynamics as a continuous system. Second, we perform an assortment of Monte Carlo simulations which corroborate that the ordinary differential equations are a good approximation of the median value. Using the solution of the ODEs, we conclude that, for medium and long-term marketing strategies, the trade-off between quality and appeal is logarithmic: The expected value of the market share remains constant if a decrease of quality is followed by an exponential increase of the product appeal. However, for very short-term marketing strategies, the trade-off is linear. The Monte Carlo simulations are then used to understand the magnitude of the stochastic fluctuations (i.e., variability) in the market share for different values of the parameters. We observe that market share variability increases as products are closer in quality and when product appeal are low. We then conclude with recommendations on marketing strategies for markets with short and long time horizons and the importance of investing early in marketing campaigns.

### 1.1 Related literature

The seminal work on the MusicLab [11] is the main motivation behind this paper. In the MusicLab experiment, participants were shown a list of songs and they were allowed to listen to any of them and then to download any song they liked. The participants were divided into two groups: the *independent* condition and the *social influence* condition. In the independent group, the songs were shown to participants in a random order. In the second group (social influence condition), the songs were displayed using the popularity ranking, i.e., the songs were displayed by decreasing number of past downloads, with the most popular songs being assigned to the more visible positions. The participants under the social influence condition were able to observe the number of downloads of each song and were divided into eight different “worlds” which evolved independently. Participants in world *i* could only observe the number of downloads per song associated with world *i*. The MusicLab experiment is a clean experimental setup for a *trial-offer market*: a market where consumers choose products (songs in the case of MusicLab) to try and potentially buy. One of the main results in [11] is that participants are strongly affected by the social influence signal and, as a consequence, different worlds evolved in dramatically different ways. In a follow-up work, Abeliuk et al [12] have conducted a similar experiment to understand the impact of different ranking policies in terms of predictability and market inequality.

Krumme et al [13] proposed a mathematical model of trial-offer markets to explain the results obtained in [11]. Their model is based on a multinomial logit choice model and incorporates product appeals and qualities, position biases, and social influence. A theoretical examination of trial-offer markets by analyzing a ranking policy that maximizes the number of expected purchases for each participant was performed by [14]. The authors proved that this greedy policy, which they called the *performance ranking*, can be computed in polynomial time and reduces drastically the market unpredictability. Another way to rank products, known as *quality ranking*, presents the products by decreasing order of their quality (the quality of a product is here defined as the probability that a consumer would purchase/download the product once she has tried the product out). Van Hentenryck et al [10] showed that the quality ranking is optimal in the long run. Moreover, the authors proved that, if a static ranking is used, the market converges to a monopoly (regardless of the ranking). The monopoly result for the long-term dynamics in [10] contrasts with the finite-time horizon, which is the focus of this paper. By considering a finite time horizon in which sales can occur, the monopoly cannot occur and it becomes interesting to understand how the market shares evolve in the short term.

The closest related work is by [15]: The authors consider a market with two competing products in which consumers arrive sequentially and purchase exactly one of them. Their model can be represented exactly as the two-product trial-offer market studied in this paper. One of the main questions the authors study is how long it would take for the highest quality product to overcome the lowest quality product forever (Note that in the long run the system converges to a monopoly for the highest quality product). They successfully answered this question by providing a probability distribution of last time *t* at which the lowest quality product had more purchases. The distribution has a very long tail, which means that an early stroke of luck at the beginning of the market provides the low quality product with an advantage that takes a very long time to overcome. They name this phenomenon as the “*struggle of the fittest*”. In a follow-up paper, [16] extended the results to a non-linear model. Our focus in this paper is different however: *We are interested in understanding which product will become the most popular as a function of the product appeals and qualities over a finite time horizon T*. Our setting is particularly appealing to model the evolution of technological products, as they typically have a short life-span and become obsolete rapidly (e.g., most cell-phone stay less than 2 years in the market). An understanding of such issues plays an important role on key strategic decisions. Indeed, firms may try to understand whether it is more advantageous to make a product of higher quality or to increase the advertising budget (i.e., to increase the product appeal).

Another related paper by [17] analyzed the effect of social influence on how consumers choose apps in the Google Play platform. In a similar vein, [18] performed experiments about how people select hotels under social influence. Both papers arrived to the same conclusion: The effect of the number of past purchases is much stronger than consumer ratings.

## 2 The model

We consider a simplification of the model proposed by [13] and later studied by [14], which consists of a market with a finite time horizon *T* in which consumers can try a product before deciding whether to buy. Consumers arrive sequentially and try only one of two competing products. If a consumer likes the product, she will buy it; Otherwise, the consumer leaves without making any purchase. Each product *i* = 1, 2 is characterized by two values:

- Its appeal *A*_*i*_ which represents inherent customer preference for trying product *i*;
- Its quality *q*_*i*_ which represents the probability that a consumer purchases product *i* after having tried *i*.

An incoming customer may try product 1 or 2. The purchase probability of product *i* (Eq 1) is given by the product of the probability *δ*_*i*_ that product *i* is chosen and the quality *q*_*i*_, i.e.,

$$\begin{array}{c} p_{1}=q_{1}\overset{\delta_{1}}{\overset{︷}{\frac{d_{1}+A_{1}}{d_{1}+A_{1}+d_{2}+A_{2}}}}\;\text{and}\;p_{2}=q_{2}\overset{\delta_{2}}{\overset{︷}{\frac{d_{2}+A_{2}}{d_{1}+A_{1}+d_{2}+A_{2}}}} \end{array}$$ (1)

where *d*_*i*_ is the number of purchases product *i* has at the time the incoming consumer arrives. We assume that *q*_1_ ≠ 0 and *q*_2_ ≠ 0.

Without loss of generality, this paper presents the results from the point of view of firm 2 whose product has the lowest quality. Hence it is only necessary to analyze how the parameters of product 2 shape its success in the market. The success is measured by the *market share* of product 2, i.e., *MS*_2_ ≐ *d*_2_/(*d*_1_ + *d*1_2_).

## 3 The ordinary differential equations model

We begin by studying the dynamics of the cultural market behavior in the transitory regime using a continuous approximation, which is modeled as a system of Ordinary Differential Equations (ODEs). The use of ODEs for solving discrete systems is well-established [19] and provides us with a battery of mathematical tools from dynamics systems theory [20]. Under this approach, the number of purchases is represented as a continuous quantity whose derivative is proportional to its quality. Obviously, this approximation of the market dynamics becomes more accurate in a system in which the number of purchases is high enough so that the fluctuations due to the discrete and probabilistic nature of the purchases becomes negligible.

By taking the interval between two consecutive customer arrivals as the time unit, the change in purchases at time *t* for the two products is given by their purchase probability:

$$\frac{d\;d_{1}}{dt}=q_{1}\frac{d_{1}+A_{1}}{d_{1}+A_{1}+d_{2}+A_{2}}=p_{1}$$ (2)

$$\frac{d\;d_{2}}{dt}=q_{2}\frac{d_{2}+A_{2}}{d_{1}+A_{1}+d_{2}+A_{2}}=p_{2}$$ (3)

with

$$\begin{array}{c} d_{1}\left(t=0\right)=d_{2}\left(t=0\right)=0. \end{array}$$ (4)

This dynamical system can be simplified by redefining the temporal and purchase variables. Let *τ* ≐ *q*_1_*t*, $d_{i}^{\text{ef}}≐d_{i}+A_{i}$ (where superscript *ef* stands for *effective purchases*), and *Q*_2_ ≐ *q*_2_/*q*_1_, where *Q*_2_ denotes the quality ratio. The dynamical system can be rewritten as:

$$\frac{d\;d_{1}^{ef}}{d\tau }=\frac{d_{1}^{ef}}{d_{1}^{ef}+d_{2}^{ef}}$$ (5)

$$\frac{d\;d_{2}^{ef}}{d\tau }=Q_{2}\frac{d_{2}^{ef}}{d_{1}^{ef}+d_{2}^{ef}}$$ (6)

with

$$\begin{array}{c} d_{1}^{\text{ef}}\left(\tau =0\right)=A_{1}\;\text{and}\;d_{2}^{\text{ef}}\left(\tau =0\right)=A_{2}. \end{array}$$ (7)

This change of variables gives us some insight on the role of each parameter in the model.

- The dynamical behavior is only determined by the quality ratio *Q*_2_, while the magnitudes of *q*_1_ and *q*_2_ only affect the time scale.
- The system dynamics does not depend explicitly on the appeals (*A*_1_ and *A*_2_), whose effects are incorporated only in the initial conditions. Thus, from a dynamical standpoint, the effects of the appeals are equivalent to those of the initial purchases. This provides a natural interpretation of the role of the appeals in terms of the equivalent initial purchases (see, e.g., [10]).

Eqs 5 and 6 can be rewritten in terms of *δ*_2_ and $d_{T}^{\text{ef}}≐d_{1}+d_{2}+A_{1}+A_{2}$ (see S1 File), i.e.,

$$\frac{d\;\delta_{2}}{d\tau }=\frac{Q_{2}-1}{d_{T}^{ef}}\delta_{2}\left(1-\delta_{2}\right)$$ (8)

$$\frac{d\;d_{T}^{ef}}{d\tau }=1-\delta_{2}+Q_{2}\delta_{2}$$ (9)

where

$$\begin{array}{c} \delta_{2}\left(\tau =0\right)=\frac{A_{2}}{A_{1}+A_{2}}\;\text{and}\;d_{T}^{\text{ef}}\left(\tau =0\right)=A_{1}+A_{2}. \end{array}$$ (10)

Note that $d_{T}^{\text{ef}}$ monotonically increases in time and, when *Q*_2_ ≠ 1, Eq 8 has 2 fixed points, one stable and one unstable. When *Q*_2_ > 1 (i.e., *q*_2_ > *q*_1_), *δ*_2_ = 1 is a stable fixed point and *δ*_2_ = 0 is unstable ([21]). Hence, unless *δ*_2_(*τ* = 0) = 0, the system evolves towards *δ*_2_ = 1: As time goes to infinity, the increasing number of purchases makes the *appeals* become negligible and produces *MS*_2_ ≈ *δ*_2_. When *Q*_2_ < 1 (i.e., *q*_2_ < *q*_1_), *δ*_2_ = 0 becomes the stable state and the system evolves towards *MS*_2_ = 0. As shown by [10], the product of highest quality captures the entire market asymptotically.

Using the chain rule, Eqs 8 and 9 can be merged into one differential equation that describes the dynamics of *δ*_2_ as a function of $d_{T}^{\text{ef}}$ (see S1 File). This differential equation has an analytical solution:

$$\begin{array}{c} \frac{{\left(1-\delta_{2}\right)}^{Q_{2}}}{\delta_{2}}=\frac{A_{1}^{Q_{2}}}{A_{2}}{\left(d_{T}^{\text{ef}}\right)}^{1-Q_{2}}. \end{array}$$ (11)

In order to study the role of *qualities* and *appeals* in the *market share* transitory states, we rewrite Eq 11 to relate *MS*_2_ with the system parameters for a given *d*_*T*_ ≐ *d*_1_ + *d*_2_. By writing *δ*_2_ as a function of *MS*_2_ and $d_{T}^{\text{ef}}$ as a function of *d*_*T*_, we can compute *MS*_2_ as a function of *Q*_2_ and *A*_2_ for different values of *A*_1_ and *d*_*T*_.

**Fig 1:**
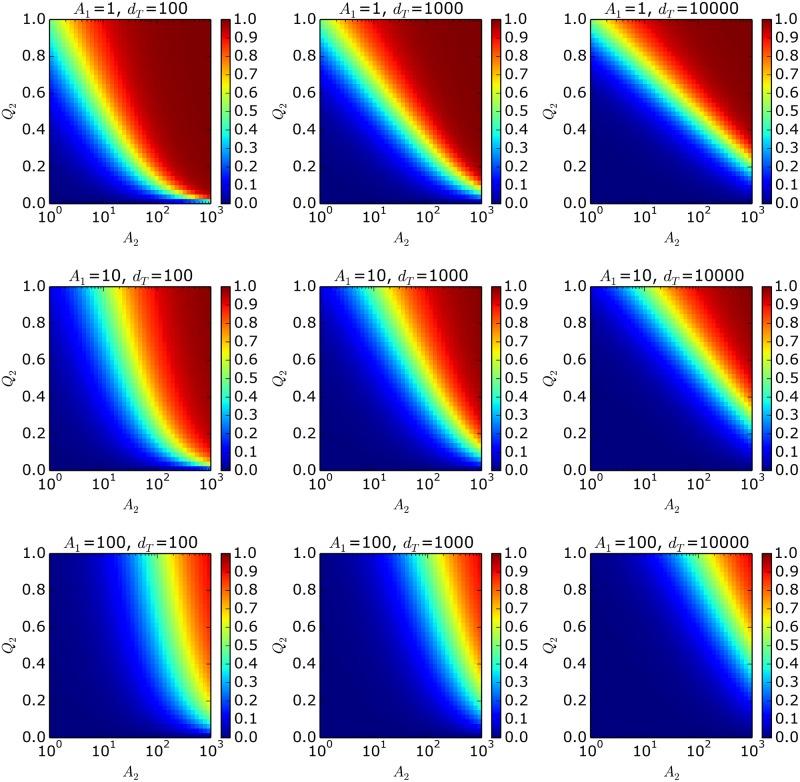
Market share of product 2 (*MS*_2_) as a function of *Q*_2_ and *A*_2_, for different values of *A*_1_ and $d_{T}^{t}$, assuming *q*_1_ = 1.

We analyze the case in which product 1 has a better quality than product 2, i.e., *Q*_2_ = *q*_2_/*q*_1_ < 1. Although product 1 is the winner (*MS*_2_ ≈ 0) in the stationary solution, at finite times, there are some combinations of parameters in which product 2 is the winner (i.e., *MS*_2_ ≈ 1), even for cases with large *d*_*T*_ values. Low *Q*_2_ values can be compensated with *A*_2_ values that are much higher than *A*_1_. It can be seen in Fig 1 that, for high *d*_*T*_ values (i.e., large number of customers), the values for *MS*_2_ shows an approximate linear behavior between *Q*_2_ and log(*A*_2_) (see the green dividing stripes in the right column). In other words, to compensate for an increment of quality for product 1, it is necessary to either increment *q*_2_ at the same rate or improve *A*_2_ by an exponential amount.

### 3.1 The long-term approximation

To study the trade-off between *Q*_2_ and *A*_2_, we can analyze the functional form of the *MS*_2_ = 0.5 level curves. These curves are displayed in Fig 1 as green stripes that separate the winning regions (in red) from the losing regions (in blue). When the time horizon is large enough to make the appeals negligible in comparison with the purchases, *MS*_2_ ≈ *δ*_2_ and $d_{T}^{\text{ef}}\approx d_{T}=d_{1}+d_{2}$. Under this approximation, the following relation can be derived (see S1 File):

$$\begin{array}{c} Q_{2}={\left(\text{ln}\left(\frac{d_{T}}{2A_{1}}\right)\right)}^{-1}\left(\text{ln}\left(d_{T}/2\right)-\text{ln}\left(A_{2}\right)\right)=\frac{\text{ln}\left(d_{T}/2\right)-\text{ln}\left(A_{2}\right)}{\text{ln}\left(d_{T}/2\right)-\text{ln}\left(A_{1}\right)}. \end{array}$$ (12)

This equation explicitly shows that, for long time periods, the green dividing strip is characterized by a linear function with a negative slope between *Q*_2_ and ln(*A*_2_), as observed in Fig 1.

### 3.2 The short-term approximation

For short time horizons, if both appeals are large (*d*_1_ ≪ *A*_1_ and *d*_2_ ≪ *A*_2_), the purchase probabilities described in Eq 1 can be approximated by

$$\begin{array}{c} p_{1}\approx q_{1}\frac{A_{1}}{A_{1}+A_{2}}\;\text{and}\;p_{2}\approx q_{2}\frac{A_{2}}{A_{1}+A_{2}} \end{array}$$ (13)

and the market share for product 2 remains constant and given by

$$\begin{array}{c} {\text{MS}}_{2}\approx \frac{\frac{Q_{2}A_{2}}{A_{1}}}{1+\frac{Q_{2}A_{2}}{A_{1}}}. \end{array}$$ (14)

The explicit dependency on $Q_{2}\frac{A_{2}}{A_{1}}$ shows that the appeals and qualities have the same impact on the market share. Thus, to compensate an increase of the rival product quality (*q*_1_), we can choose to increase our product quality or appeal linearly by the exact same rate (since *Q*_2_ = *q*_2_/*q*_1_). In this case, the curve that separates the winning and losing regions (level set of *MS*_2_ = 0.5) is

$$\begin{array}{c} Q_{2}=\frac{A_{1}}{A_{2}} \end{array}$$ (15)

Over the dividing strip, *Q*_2_ decreases inversely with *A*_2_ and not with its logarithm. Thus, the relationship between the appeal and quality of different products is linear. This approximation holds as long as *d*_1_ ≪ *A*_1_ and *d*_2_ ≪ *A*_2_. Hence large appeal values result in longer time lags during which *MS*_2_ does not evolve and Eq 14 holds.

## 4 Stochastic simulations

In the previous section, we used a deterministic model to gain some insight into the dependencies between system parameters for finite times. We now employ Monte Carlo simulations to study the effects of the stochastic fluctuations on the market share and their dependency on the system parameters. Additionally, we examine how well the ODE model approximates the real dynamic system.

**Fig 2:**
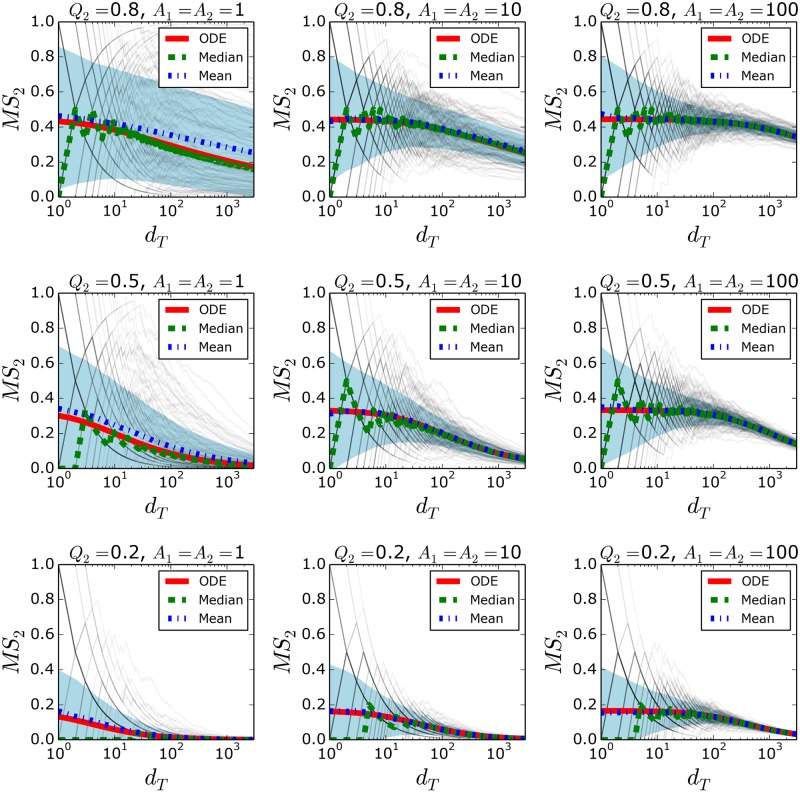
Monte Carlo simulations and ODE solutions of the market shares for symmetric appeals. The Monte Carlo simulations involved 10^4^ realizations of the system for nine different set of parameters (grey lines). In all cases we used *q*_1_ = 1, the appeal used for both products were the same (*A*_1_ = *A*_2_) and both products start with zero purchases, *d*_1_(*t* = 0) = *d*_2_(*t* = 0) = 0. Although the Monte Carlo simulations produce discrete dots in the (*d_T_*, *MS*_2_) space, we plot each simulation with straight lines that link consecutive dots to follow trajectories easily.

Fig 2 reports on Monte Carlo simulations of *MS*_2_ versus *d*_*T*_ of the discrete system and their ODE approximations for different values of *qualities* and *appeals*. The system shows large variability for small *appeals* and similar *qualities* (*Q*_2_ ∼ 1). In particular, for the case of *A*_1_ = *A*_2_ = 1 and *Q*_2_ = 0.8, product 2 obtains more purchases than product 1 in 20% of the simulations at *d*_*T*_ = 1000, despite the fact that the expected market share of product 2 at this *d*_*T*_ is *MS*_2_ = 0.28). Obviously, the system possesses a positive feedback mechanism in which the purchase of a product increases its future purchase probability. A stochastic fluctuation in favor of one product increases its purchase probability, which amplifies this initial fluctuation. However, when the appeals are larger, a substantial reduction in variability is observed. This is consistent with the fact that large appeals produce longer time lags in which the purchase probabilities do not depend on the number of purchases (see Section 3.2). This drastically reduces the positive feedback effect and diminishes the system variability. Larger appeal values result in longer time lags, which allow the system to reach high *d*_*T*_ values under the long-term approximation. Furthermore, as *d*_*T*_ increases, a small stochastic fluctuation has proportionally less influence on *d*_*T*_, producing negligible variations in the purchase probabilities. Thus, stochastic fluctuations at short times are the major contributor to the final variability. Fig 2 shows that individual trajectories become less sensitive to stochastic fluctuations as the *d*_*T*_ increases.

Fig 2 also shows that variability decreases with a decrease in *Q*_2_. Low expected values of *MS*_2_ tend to be associated with less variability. The higher the purchase probability difference between the products, the more skewed and narrow the initial distribution is. This is discussed in more detail in the Section 4.1. In the case of *Q*_2_ = 0.2, when moving from *A*_1_ = *A*_2_ = 10 to *A*_1_ = *A*_2_ = 100, the variability increases slightly, which is the opposite behavior compared to the *Q*_2_ = 0.8 case. Although the increase in the time lag tends to decrease the variability, it also delays the increase in *MS*_2_. The distribution of *MS*_2_ thus stays closer to the values of *MS*_2_ = 0.5, where the system is more susceptible to fluctuations.

**Fig 3:**
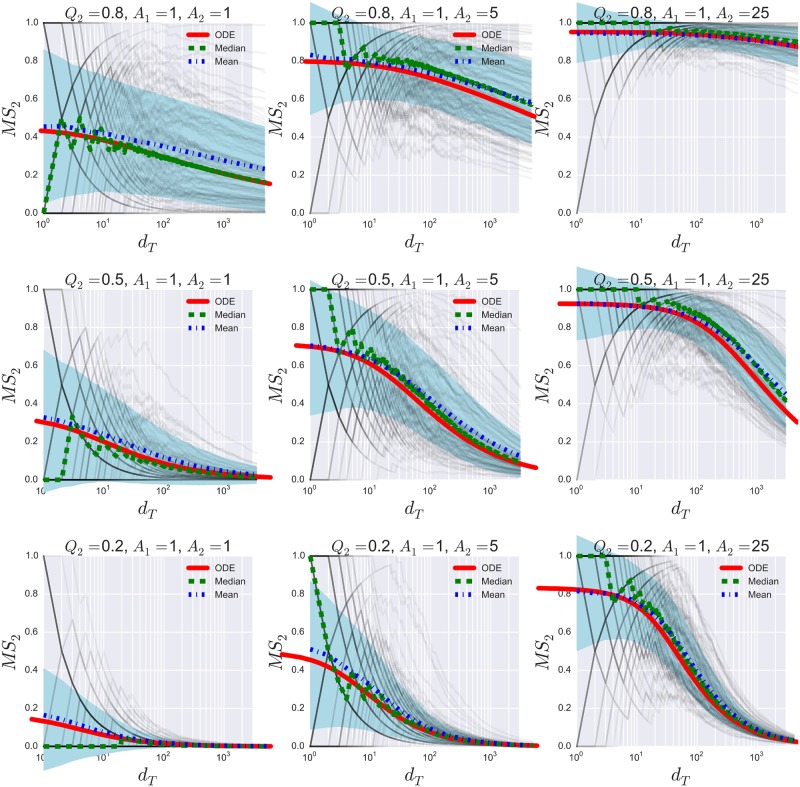
Monte carlo simulations and ODE solutions of the market shares for asymetric appeals. The Monte Carlo simulations involved 10^4^ realizations of the system for nine different sets of parameters (grey lines). In all cases, *q*_1_ = 1 and both products start with zero purchases, i.e., *d*_1_(*t* = 0) = *d*_2_(*t* = 0) = 0. Although Monte Carlo simulations produce discrete dots in the (*d_T_*, *MS*_2_) space, we plot each simulation with straight lines that link consecutive dots to follow trajectories easily.

As mentioned in Section 3, when *A*_2_ > *A*_1_, there are regions in the parameter space (*Q*_2_, *A*_2_) in which product 2 obtains a substantial market share at finite times. Fig 3 depicts this behavior with Monte Carlo simulations. It is worth noting how small changes in appeals (from *A*_2_ = 1 to *A*_2_ = 5) produce large effects sustained over time, which again can be explained in terms of a cumulative advantage process.

### 4.1 Standard deviation of the market share

By Lemma 1 in [10], the probability that product *i* is purchased next (after any number of trials) is given by

$$\begin{array}{c} {\hat{p}}_{i}=\frac{q_{i}\left(A_{i}+d_{i}\right)}{q_{1}\left(A_{1}+d_{1}\right)+q_{2}\left(A_{2}+d_{2}\right)}. \end{array}$$ (16)

Then, under the short-term approximation *d*_1_ ≪ *A*_1_ and *d*_2_ ≪ *A*_2_, the next purchase probabilities for products 1 and 2 are given by

$$\begin{array}{c} {\hat{p}}_{1}=\frac{A_{1}}{A_{1}+Q_{2}A_{2}}\;\text{and}\;{\hat{p}}_{2}=\frac{Q_{2}A_{2}}{A_{1}+Q_{2}A_{2}}. \end{array}$$ (17)

The probability of having exactly *k* purchases of product 2 given that there is a total of *d*_*T*_ purchases is a binomial distribution:

$$\begin{array}{c} \text{Prob}\left(d_{2}\left(d_{T}\right)=k\right)=\left(\begin{array}{c} d_{T}\\ k \end{array}\right){\left(\frac{Q_{2}A_{2}}{A_{1}+Q_{2}A_{2}}\right)}^{k}{\left(\frac{A_{1}}{A_{1}+Q_{2}A_{2}}\right)}^{d_{T}-k}. \end{array}$$ (18)

It follows that the variance and standard deviation for the market share of product 2 are given by

$$\begin{array}{c} \text{Var}({\text{MS}}_{2}\left(d_{T}\right))=\frac{1}{d_{T}^{2}}\text{Var}(d_{2}\left(d_{T}\right))=\frac{1}{d_{T}}(\frac{Q_{2}A_{2}}{A_{1}+Q_{2}A_{2}})(\frac{A_{1}}{A_{1}+Q_{2}A_{2}}), \end{array}$$ (19)

$$\begin{array}{c} \sigma_{{\text{MS}}_{2}\left(d_{T}\right)}=\frac{1}{\sqrt{d_{T}}}(\frac{\sqrt{Q_{2}A_{2}A_{1}}}{A_{1}+Q_{2}A_{2}}). \end{array}$$ (20)

This approximation holds as long as *d*_1_ ≪ *A*_1_ *d*_2_ ≪ *A*_2_ and Eq 20 shows that larger appeals result in longer time lags in which $\sigma_{{\text{MS}}_{2}\left(d_{T}\right)}$ decrease proportionally to $1/\sqrt{d_{T}}$ (see Fig 2 for an illustration). Additionally, the second factor reaches its maximum when *A*_1_ = *A*_2_*Q*_2_, which coincides with the case where the expected market shares of the products are equal. This second factor also explains the reduction of variability when *Q*_2_ is low, which was observed in Fig 2.

### 4.2 The quality of the ODEs approximation

Under the short-term approximation, when appeals are large, the mean *MS*_2_ value is ${\hat{p}}_{2}$ (computed from the purchase binomial distribution in Eq 18), which coincides with the ODE solution (Eq 14). This is due to the linear dependencies of the purchase probabilities under the short-term approximation (Eq 17). However, when *d*_*T*_ increases, the non-linearities of the purchase probabilities can produce a discrepancy between the ODE solution and the mean value of the simulation. Fig 2 shows that, in general, the mean values of the simulations are in strong agreement with the ODEs solution. Nevertheless, when *A*_1_ = *A*_2_ = 1 and *Q*_2_ = 0.8, there is a difference between them. This is consistent with the fact that, when the *appeals* are large, the short-term approximation holds for longer *d*_*T*_ ranges and hence the mean value and the ODE match during the times when the system is most susceptible to stochastic fluctuations. Note also that the median of the simulations seems to be in strong agreement with the ODE and to outperform the mean. However, for very short times, it fails to reproduce the ODE solution, which is expected since the median is restricted to the possible discrete values of the simulations. For example, if the ODE gives a *MS*_2_ = 0.2 for *d*_*T*_ = 2 there are only three possible combinations of (*d*_1_, *d*_2_) at this time; {(2, 0), (1, 1), (0, 2)}. These correspond to only three possible *MS*_2_ values, {1, 0.5, 0}, which would not match the ODE solution.

## 5 Discussion and conclusions

This paper studied the transitory dynamics for a simple trial-offer market model, originally proposed in [13], where consumer choices are dependent on the product appeals (*A*_*i*_), past purchases (*d*_*i*_), and product quality (*q*_*i*_). In this model, consumers arrive sequentially, select a product to try, and then decide whether to purchase the sampled product. Although the asymptotic convergence or stationary states of these and related dynamical systems has been studied in depth, the short-term dynamics remained relatively unexplored. More precisely, the asymptotic convergence of the trial-offer market studied here was solved by [10] who proved that the product with the highest quality will capture the entire market in the limit when *T* goes to infinity. However, the impact of product quality and appeal on the market shares for a finite time horizon has not been studied.

To analyze this finite-time behavior, the paper modeled the discrete market dynamics as a system of Ordinary Differential Equations (ODEs) and restricted attention to two products for simplicity. From an ODE nondimensionalization, the paper showed that: (1) The dynamical behavior is only determined by the quality ratio (*Q*2 ≐ *q*_2_/*q*_1_); and (2) The effect of the appeals is equivalent to those of the initial purchases. This provides a natural interpretation of the role of the appeals in terms of the equivalent initial purchases.

Moreover, to analyze possible marketing strategies, the paper considered a firm whose product (product 2) is of a lower quality than the product of its competitor (product 1). The paper solved the ODEs for different parameter sets and quantified the market share at different time points. Although product 1 is the winner asymptotically, the paper showed that, at finite times, there are parameter regions in which product 2 obtains a higher market share, even in the case of long time horizons. In other words, *a low quality can be compensated with a large appeal*, *which can be seen as the result of a significant advertising campaign*.

The analytic solution of the ODEs also allowed us to identify the impact of the system parameters on the market shares. In particular, the paper identified two limit cases for which we can propose different market strategies: (1) the long-term strategy, in which the appeals are negligible in comparison to the total purchases (*A*_1_, *A*_2_ ≪ *d*_*T*_) and (2) The immediate reward strategy, in which the time horizon is short enough that the number of purchases are negligible in comparison to the appeals. For the long-term strategy, the paper showed that the trade-off between the appeals and qualities lies in a logarithmic scale for the appeals and in a linear scale for the qualities. *For long-term success, it is thus highly beneficial to improve the quality of product 2*, *since the marketing cost is likely to become prohibitive*. *For short time horizons, the paper showed that the appeals and qualities have the same impact on the market share*. In this case, firm 2 has the choice of improving either the appeal or the quality of product 2.

Finally, the paper ran Monte Carlo simulations to study the effect of the stochastic fluctuations on the market share. The results indicate a good agreement between the ODEs solutions and the median and mean of the simulations for a variety of parameter settings. The results also showed that the system exhibits large variability when both appeals are small and their qualities are similar. These observations have once again important implications on potential marketing strategies. Indeed, since the system has an implicit positive feedback, in which the purchase of a product increases its future purchase probability, *an immediate reward strategy should focus on improving the appeal of product 2 at the expense of its quality in order to eliminate the positive feedback of initial fluctuations*. More generally, the results show that large appeals reduce the system variability and improve the predictions of the ODE model.

In summary, the paper provides evidence that (1) early investments in advertising are highly beneficial for short time horizons and for improving market predictibility; and (2) quality improvements are desirable for long-term strategies.

## Supporting information

S1 File Supplementary materials. (PDF)
