# Supplementary material for: Transient dynamics in trial-offer markets with social influence: Trade-offs between appeal and quality
Source: PLoS One. 2017 Jul 26;12(7):e0180040. doi: 10.1371/journal.pone.0180040 (PMC5528888; doi:10.1371/journal.pone.0180040)
Supplement: S1 File — (PDF) [file pone.0180040.s001.pdf]

1 Supplementary Materials

1.1 Analytic Solution

Consider a system of two products. At time t, the probability of purchasing products 1 and 2 is given by

$$\frac{d\,d_1}{dt} = q_1 \frac{d_1 + A_1}{d_1 + A_1 + d_2 + A_2} \tag{1}$$

$$\frac{d\,d_2}{dt} = q_2 \frac{d_2 + A_2}{d_1 + A_1 + d_2 + A_2} \tag{2}$$

with

$$d_1(t = 0) = d_2(t = 0) = 0. \tag{3}$$

Moreover, this dynamical system can be rewritten into

$$\frac{d\,d_1^{ef}}{d\tau} = \frac{d_1^{ef}}{d_1^{ef} + d_2^{ef}} \tag{4}$$

$$\frac{d\,d_2^{ef}}{d\tau} = Q_2 \frac{d_2^{ef}}{d_1^{ef} + d_2^{ef}} \tag{5}$$

with

$$d_1^{ef}(\tau = 0) = A_1 \text{ and } d_2^{ef}(\tau = 0) = A_2. \tag{6}$$

By denoting $Q_2 \doteq q_2/q_1$, we can redefine the temporal variable as $\tau = q_1 t$ and the independent variables as $d_i^{ef} = d_i + A_i$. Using the notations

$$d_T^{ef}(t) \doteq d_1^{ef} + d_2^{ef} \text{ and } \delta_2(t) \doteq \frac{d_2^{ef}}{d_T^{ef}}, \tag{7}$$

it follows that

$$\frac{d\,d_1^{ef}}{d\tau} = 1 - \delta_2 \text{ and } \frac{d\,d_2^{ef}}{d\tau} = Q_2\delta_2, \tag{8}$$

$$\frac{d\,d_T^{ef}}{d\tau} = \frac{d\,d_1^{ef}}{d\tau} + \frac{d\,d_2^{ef}}{d\tau} = 1 - \delta_2 + Q_2\delta_2 \tag{9}$$

$$\Rightarrow \frac{d\,\delta_2}{d\tau} = \frac{\frac{d\,d_2^{ef}}{d\tau} d_T^{ef} - \frac{d\,d_T^{ef}}{d\tau} d_2^{ef}}{(d_2^{ef})^2} = \frac{Q_2\delta_2 d_T^{ef} - \delta_2 d_T^{ef}\left(Q_2\delta_2 + (1 - \delta_2)\right)}{(d_T^{ef})^2}. \tag{10}$$

We obtain a new set of differential equations

$$\frac{d\,\delta_2}{d\tau} = \frac{Q_2 - 1}{d_T^{ef}} \delta_2 \left(1 - \delta_2\right), \tag{11}$$

$$\frac{d\,d_T^{ef}}{d\tau} = 1 - \delta_2 + Q_2\delta_2 \tag{12}$$

where

$$\delta_2(\tau = 0) = \frac{A_2}{A_1 + A_2} \tag{13}$$

$$d_T^{ef}(\tau = 0) = A_1 + A_2. \tag{14}$$

We can merge Eqs 11 and 12 in a single differential equation for the dynamics of δ_2 in terms of d_T^{ef}

$$\frac{d\,\delta_2}{d\,d_T^{ef}} = \frac{d\,\delta_2}{d\tau}\frac{d\tau}{d\,d_T^{ef}} = \frac{(Q_2-1)\delta_2\,(1-\delta_2)}{d_T^{ef}\,(1-\delta_2+Q_2\delta_2)} \tag{15}$$

with the initial condition

$$\delta_2(d_T^{ef} = A_1 + A_2) = \frac{A_2}{A_1+A_2}. \tag{16}$$

Lemma 1. A general solution to the ODE in Eq 15 is given by

$$C + (1-Q_2)\log(d_T^{ef}) = Q_2\log(1-\delta_2) - \log(\delta_2), \tag{17}$$

where C is an integration constant.

Proof. Deriving both sides of Eq 17 respect to d_T^{ef} we obtain

$$(1-Q_2)\frac{1}{d_T^{ef}} = Q_2\frac{1}{1-\delta_2}\frac{d\delta_2}{dd_T^{ef}} - \frac{1}{\delta_2}\frac{d\delta_2}{dd_T^{ef}}, \tag{18}$$

and by rearranging the equation we get Eq 15. □

As a simplification, we may write Eq 17 as

$$\tilde{C}d_T^{ef\,(1-Q_2)} = \frac{(1-\delta_2)^{Q_2}}{\delta_2}, \tag{19}$$

where $\tilde{C} \doteq \exp(C)$. Now we know that Eq 19 is a solution to our ODE, so we must impose the initial conditions to obtain a final solution. This is

$$\tilde{C}(A_1+A_2)^{(1-Q_2)} = \frac{\left(\frac{A_1}{A_1+A_2}\right)^{Q_2}}{\frac{A_2}{A_1+A_2}}, \tag{20}$$

so

$$\tilde{C} = \frac{A_1^Q}{A_2}. \tag{21}$$

Finally, plugging in Eq 21 in Eq 19, we obtain

$$\frac{(1-\delta_2)^{Q_2}}{\delta_2} = \frac{A_1^{Q_2}}{A_2}(d_T^{ef})^{1-Q_2}. \tag{22}$$

Since $\delta_2 = (MS_2\,d_T + A_2)/d_T^{ef}$, Eq 22 can be rewriten into

$$\frac{\left(d_T^{ef} - MS_2\,d_T - A_2\right)^{Q_2}}{MS_2\,d_T + A_2} = \frac{A_1^{Q_2}}{A_2}. \tag{23}$$

1.2 Long-Term Approximation

When the time horizon is large enough to make the appeals negligible in comparison to the total purchases, $MS_2 \approx \delta_2$ and $d_T^{ef} \approx d_T$, with $d_T = d_1 + d_2$. In particular, the curve that separates the winning and losing regions in the parameter space can be obtained by setting $M_2 = 0.5$ in Eq 22 to obtain

$$\frac{(0.5)^{Q_2}}{0.5} = \frac{A_1^{Q_2}}{A_2}(d_T)^{1-Q_2} \tag{24}$$

which can be rewritten as

$$Q_2 = -\ln^{-1}\left(\frac{d_T}{2A_1}\right)(\ln(A_2) - \ln(d_T/2)) = \frac{\ln(d_T/2) - \ln(A_2)}{\ln(d_T/2) - \ln(A_1)}. \tag{25}$$
